# Editorial

**DOI:** 10.1155/EDR.2003.i

**Published:** 2003


*Experimental Diab. Res.*, 4:i, 2003
Copyright © Taylor and Francis Inc.
ISSN: 1543-8600 print / 1543-8619 online
DOI: 10.1080/15438600390239502

### Editorial

The International Journal of Experimental Diabetes Research concluded its third year of publication in 2002. We wish to thank contributing authors, reviewers, as well as the publisher, Taylor and Francis, for making this publication a successful newcomer in the arena of diabetes research.

As is well known to scientists and clinicians alike, and increasingly to the general public, the epidemics of diabetes and obesity are upon us and the prospects for the future look bleak. This modern plague is and will be associated with an increasing number of suffering patients and with increasing economic pressure on governmental and private health care delivery organizations and on society as a whole.

There is no immediate remedy for these epidemics. Rather, we are faced with long-term prospectives of intensifying primary and secondary prevention of Type 2 diabetes and of the closely related risk factor posed by obesity. Last but not least, intensified basic research into risk factors, biochemical, molecular, and genetic predispositions to the development of diabetic and related disorders will be paramount.

Facing the serious prospects of these interrelated global epidemics, the editorial board of The International Journal of Experimental Diabetes Research has expanded its scope, by inviting publications on basic obesity research and on its interrelationships with diabetes research. To reflect this expansion of the scope of the Journal, we have renamed it EXPERIMENTAL DIABESITY RESEARCH, which started with the first issue of 2003. Furthermore, with the increasing submission of high quality contributions, the Journal shall publish six issues per year, hopefully starting from 2004. With the increasing scientific coverage of the renamed journal, we announce the expanded editorial board, which now includes international experts in the field of obesity research.

We hope that this widening horizon of the Journal will provide an efficient and effective vehicle in distributing high quality research relevant to the understanding and eventually a curtailment of the global epidemic of “diabesity.”

Anders A. F. Sima and Eleazar Shafrir

